# Parameters of ovarian reserve in relation to urinary concentrations of parabens

**DOI:** 10.1186/s12940-020-00580-3

**Published:** 2020-03-02

**Authors:** Joanna Jurewicz, Michał Radwan, Bartosz Wielgomas, Anetta Karwacka, Anna Klimowska, Paweł Kałużny, Paweł Radwan, Wojciech Hanke

**Affiliations:** 1 Department of Environmental Epidemiology, Nofer Institute of Occupational Medicine, 8 Teresy St, 91-362 Lodz, Poland; 2 Department of Gynecology and Reproduction, “Gameta” Hospital, 34/36 Rudzka St, 95-030 Rzgów, Poland; 3 Faculty of Health Sciences, The State University of Applied Sciences in Plock, 2 Dabrowskiego Sq, 09-402 Plock, Poland; 4 Department of Toxicology, Medical University of Gdańsk, 107 Hallera St, Gdańsk, Poland; 5 Gyncare Clinic, 7 Chodkiewicz St, 02-593 Warsaw, Poland

**Keywords:** Ovarian reserve, Parabens, Fertility, Environmental exposure

## Abstract

**Background:**

Parabens are synthetic chemicals commonly used in cosmetics, pharmaceuticals, food and beverage processing as antimicrobial preservatives. In experimental animals, parabens exposure was associated with adverse effects on female reproduction. Despite the widespread use of parabens little is known about their effect on female fecundity. The objective of the current analysis was to evaluate the associations of urinary parabens concentrations with parameters of ovarian reserve among women undergoing treatment in a fertility clinic.

**Methods:**

Five hundred eleven female aged 25–39 years who attended the infertility clinic in central region of Poland for diagnostic purposes were recruited between September 2014 and February 2019. Urinary concentrations of parabens were measured by a validated gas chromatograohy ion-tap mass spectrometry method. Parameters of ovarian reserve were: antral follicle count (AFC), a*nti-Müllerian hormone (AMH),* follicle-stimulating hormone (FSH) and estradiol (E_2_) *levels.*

**Results:**

The geometric mean of specific gravity adjusted urinary concentrations of methyl (MP), ethyl (EP), propyl (PP), butyl (BP) and izobutyl paraben (iBuP) were 107.93 μg/L, 12.9 μg/L, 18.67 μg/L, 5.02 μg/L and 2.80 μg/L. Urinary concentrations of PP in the third quartile of exposure ((50–75] percentyl) were inversely associated with antral follicle count (*p* = 0.048), estradiol level (*p* = 0.03) and positively with FSH concentration (*p* = 0.026). MP, EP, BP and iBuP parabens were not associated any with parameters of ovarian reserve.

**Conclusions:**

Chronic exposure to PP may potentially contributing to reduced fecundity and impair fertility. As this is one of the first study to investigate the potential effect of parabens on ovarian reserve further epidemiological studies with longer duration of observation are needed.

## Background

Parabens are synthetic chemicals, esters of 4-hydroxybenzoic acid widely used in cosmetics such as shampoos, moisture solutions, shaving gels, spray tanning solution, toothpaste, pharmaceuticals, food and beverage processing as antimicrobial preservatives because of their antibacterial and fungicidal properties [1]. Dermal absorption from personal care products, including lotions and cosmetics is considered to be a main route of parabens exposure [2], but exposure can occur also through ingestion and inhalation [2]. Parabens are popular because of their low toxicity and cost [3]. Parabens, estrogenic chemicals [3, 4] bind both estrogen receptor (ER)-alpha and (ER)-beta [5, 6]. The estrogenic activity of parabens increases with the length and branching of the alkyl chain [7, 8]. Chen et al. (2007) and Darbre and Harvey (2008) [9, 10] have reported that parabens may also act as antiandrogens and may affect thyroid function [8, 11].

A few animal toxicity studies have reported adverse effect of parabens on female reproductive and endocrine function [8, 11, 12]. Vo et al. (2010) [8] found that prepubertal female rats treated orally with parabens had a decrease in ovarian weight (MP, isopropyl paraben (i-PP)) and histopathological changes in the ovaries (MP, i-PP, BP, iBuP) such as a decrease in corpora lutea, an increase in number of cystic follicles and thinning of follicular cells as well as decrease serum estradiol levels. A decrease in ER-β expression in the ovaries was observed in a study evaluating pregnant rats exposed to parabens [11]. On the other hand Kang et al. (2002) [12] did not observe effect on reproductive organ weights and histopatological abnormalities in female offspring.

Human data on the female reproductive health effects of paraben exposure are limited. To date only three studies have evaluated exposure to parabens and women reproductive potentials. One study assessed the ovarian reserve [13], and two evaluated fertilization rate, embryo quality, implantation and live births [14, 15]. Smith et al. (2013) [13] observed a trend of lower AFC with increasing urinary PP level among 193 female patients from the Massachusetts General Hospital Fertility Center [13]. The next study performed in United States evaluate the associations of paternal urinary concentrations of MP, PP and BP in relation to reproductive outcomes among 218 couples from a fertility center. Decreased odds of live birth was observed only after exposure to MP [14]. On the other hand, in the study performed by Minguez-Alarcon and co-workers (2016) [15], among 245 women undergoing infertility treatments, urinary concentrations of MP, PP, BP were not associated with IVF outcomes specially total and mature oocyte count, proportion of high embryo quality, fertilization and implantation rate, clinical pregnancy and live births [15].

This study adds to the previous human studies of parabens exposure more statistical power, by larger study subjects and by assessment of exposure in two urine samples, which in case of nonpersistant chemicals is important. Additionally, exposure to five different parabens (MP, EP, PP, BP, iBu-P) were assessed.

The aim of this study was to determine whether the environmental exposure to parabens affects female ovarian reserve, one of the predictor of female fecundity among women undergoing treatment in a fertility clinic.

## Methods

### Study participants and data collection

The study population consists of 511 females aged 25–39 years attending infertility clinic in central region of Poland for diagnostic purposes, because of couples’ infertility (the failure to achieve a clinical pregnancy after 12 months or more of regular unprotected sexual intercourse) [16] between September 2014 and February 2019.

Only menstruating women who have confirmed ovulatory cycles without clinical co-existing chronic diseases that may reduce ovarian reserve (eg, adrenocortical insufficiency, abnormal karyotype, fragile X syndrome) were eligible for the study. Exclusion criteria included: spontaneous premature ovarian failure, previous surgical treatment of the ovaries, chemotherapy or pelvic radiotherapy (conditions that may lead to reduction of ovarian reserve), cysts in the ovaries including endometriates (excluding functional cysts) and conditions with no ovulatory cycles such as polycystic ovary syndrome, hypogonadotropic hypogonism, hyperprolactinemia.

The Nofer Institute of Occupational Medicine Bioethical Committee Board approved the study and written informed consent was obtained from all subjects before their participation.

All participants were interviewed and provided a blood and urine samples during their office visit. The information about demographics, socio-economic status, stress (life and occupational stress), medical history, lifestyle factors (smoking, alcohol consumption, diet, physical activity) and occupational exposures (physical and chemical exposure) were collected using nurse-administrated questionnaire at entry into the study.

### Assessment of ovarian reserve parameters

The female ovarian reserve was assessed by AFC, concentration of hormones: AMH, FSH and E_2_.

The AFC was measured in both ovaries in accordance with the recommendations of Broekmans et al. (2010) [17] only by certified specialist in the field of ultrasound in gynecology, trained in the evaluation of AFC. All tests were carried out at the beginning of the follicular phase, usually between 2 and 4 days of the cycle. Antral follicles with dimensions of 2 to 10 mm were considered for the assessment. The sum of antral follicles from the left and right ovaries was used for the analysis [18].

The intravenous blood sample was drawn and the serum was analysed for AMH with an enzyme linked immunoabsorbent method utilizing commercially available Gen-II ELISA kits according to manufacturer instruction (Beckman Coulter, Inc., USA). The FSH, and E_2_ were measured using enhanced chemiluminescence method for VITROS ECi Immunodiagnostic System with MicroWell technology utilizing commercially available VITROS Reagent Packs and the VITROS Calibrators for the hormones according to manufacturer instruction (Ortho-Clinical Diagnostics Johnson & Johnson, UK).

No fertility medications were used in the cycles proceeding assessment of ovarian reserve parameters..

### Urinary parabens measurements

A convenience urine sample was collected in sterile polypropylene cup from each subject at the time of recruitment and from subsets of the study population (*N* = 120) at subsequent visits during infertility treatment cycles. Second urine sample was collected to confirm that exposure to parabens is habitual. Women with the second urine sample collected were not different (*p* > 0.05) from the whole study population in terms of exposure to parabens, and characteristics (age, past diseases, alcohol consumption, duration of infertility) that may be associated with parameters of ovarian reserve.

After measuring specific gravity (SG) using a handheld refractometer the urine samples were frozen at − 20 °C and sent to the laboratory in Department of Toxicology, Medical University of Gdańsk. Parabens were analyzed by GC-MS/MS method. The standard stock solutions (1 mg/ml) of each paraben were prepared in acetonitrile. The stock solutions were used to prepare two separate working solutions: one for fortification of quality control urine samples and the other one for the calibration. All solutions were stored at − 20 °C in the dark and were stable for at least 6 months described by Lu et al. (2015) [19] with some modifications. The method allows to evaluate the total concentration (free plus conjugated) of analyzed parabens in urine samples and was described previously [19]. Since, the major source of parabens to humans are cosmetic products, personnel handling urine samples was instructed to avoid paraben containing personal care products during the sample collection, sample preparation and analysis to minimize the risk of external contamination.

Briefly, three milliliters of urine sample were hydrolyzed with a mixture of 1 M acetate buffer (pH 5.0) and β-glucuronidase from *Helix pomatia* type HP-2. After deconjugation procedure, parabens were extracted using n-hexane and methyl *tert*-butyl ether mixture (3:1, *v*:*v*), then the organic phase was separated and cleaned-up with PSA and anhydrous magnesium sulfate. The cleaned extract was evaporated to dryness under gentle stream of nitrogen, subsequently the dry residue was dissolved in 50 μl of BSTFA:TMCS (99:1) and derivatized for 30 min at 40 °C. One microliter of final extract was injected into GC-MS system.

Analyses were performed using gas chromatograph (Varian GC-450) equipped with low bleed VF5-ms capillary column (30 m × 0.25 mm × 0.25 μm + 10 m EZ-guard, Varian) and 1177 split/splitless injector (isothermal condition: 280 °C). The oven column program was: 60 °C (3 min), 60 °C–140 °C (120 °C/min), 140 °C–290 °C (17 °C/min), 280 °C (13 min). Tandem mass spectrometry (ion trap mass spectrometer, Varian 220-MS) was applied as detection method. For quantitative analysis the following precursor and daughter *m/z* ions were used: 224 and 209, 177 for MP; 238 and 195, 209, 223 for EP; 210 and 195 for PP, iBuP as well as BP. Labeled analogs of MP, PP and BP were used as internal standards with ion transitions *m/z*: 228 to 213, 197 and 180 for MP d4; 216 to 210 for PP ^13^C_6_ and BuP ^13^C_6_. The underlined ions were utilized for quantitative analysis. We used urine fortified at three concentration levels: low (LQC), medium (MQC) and high (HQC) for in-house quality control of the analytical procedure. Daily analytical batch consisted of one blank urine sample, two LQC and MQC samples, one HQC sample, 40 real urine samples and two reagent blank samples (all reagents without urine) to control external contamination during sample processing. The between-day precision was below 20% for all studied parabens. Westgard rules were applied to validate each batch.

The applied analytical procedure was fully validated during developing process. However, when series of real samples were analyzed validation was limited to calibration curve preparation and evaluation of within- and between-day precision before processing target urine samples. The calibration curve was prepared as sequential dilution of working solution in the range of 0.5–1000 ng/ml. Samples which concentration exceeded the highest point of calibration curve were reanalyzed using smaller volume of urine. The limit of detection for MP, EP and PP was 0.5 ng/ml and for iBuP and BP 1.0 ng/ml [20]. The within- and between-day imprecision was 20 and 8% for MP and EP and 5% for PP, iBuP and BP, respectively.

### Statistical analysis

Descriptive statistics for subjects grouped by demographic characteristics were calculated, along with the distributions of urinary parabens, and antral follicle count and reproductive hormone levels. Spearman correlation coefficient was used as nonparametric measure of associations between concentrations of different parabens. Multiple least squares linear regression models were used to quantify the associations of urinary parabens (explanatory variables) with antral follicle count and the concentrations of reproductive hormones as dependent variables.

Parabens concentrations <LOD were assigned a value equal to LOD divided by the square root of two [21]. Urinary parabens concentrations (μg/L) were adjusted for SG (specific gravity) using the formula: Pc = P[(1.016–1)/(SG-1)], where Pc is the SG-corrected paraben concentration (μg/L), P is the measured paraben concentration, and 1.016 is the median SG (specific gravity of the urine sample) level in the study population.

MP, EP, PP, BP were categorized into 4 groups, first one consisted of values below limit of detection (LOD) to 25th percentile value, second-greater than the 25th percentile value to the median, third greater than the median to 75th percentile value, while the fourth group consisted of values greater than the 75th percentile. Additionally urinary concentrations of those parabens were presented as a continuous variables. Due to the high proportion of samples below the LOD in case of iBuP paraben concentrations were categorized as above and below LOD.

Multivariable linear regression was used to explore a relationship between urinary paraben concentrations and hormone levels and antral follicle count.

Inclusion of covariates in the multivariable regression models was based on biological and statistical consideration. The following covariates were considered as potential confounders: age (years), smoking (yes/no), BMI (kg/m^2^), initial infertility diagnosis. R statistical software (ver.3.5.1) was used for analysis [22].

## Results

### Study population characteristics

Demographic characteristics of the study participants are presented in Table 1. In summary, most women had higher (75.34%) or secondary (21.14%) education and were non smokers (92.17%). The mean (±SD) age and body mass index (BMI) were 33.30 ± 3.69 years and 23.18 ± 3.80 kg/m^2^ respectively. Most of the study females drank none or less than 1 drink per week (55.0%). Duration of couple’s infertility lasted mostly > 5 years (35.23%) and 3–5 years (29.55%). The initial infertility diagnosis was mostly male factor (37.8%), followed by idiopathic infertility (31.1%) and female factor (28.57%) (Table 1).

**Table 1:** Characteristics of the study population *N* = 511

Variables	N (%)
Education	
Vocational	18 (3.52)
Secondary	108 (21.14)
Higher	385 (75.34)
Age [years]	
24–30	121 (23.68)
31–39	390 (76.32)
Mean ± SD	33.30 ± 3.69
BMI [kg/m^2^]	
< 18,5	29 (5.68)
18,5-24,9	301 (58.90)
25–29,9	154 (30.14)
30–40	27 (5.28)
Mean ± SD	23.18 ± 3.80
Smoking	
No	471 (92.17)
Yes	40 (7.83)
Initial infertility diagnosis	
Male factor	193 (37.8)
Idiopathic	159 (31.1)
Female factor	146 (28.6)
Ovulation disorders	62 (12.1)
Tubal factor	24 (4.7)
Uterine factor	8 (1.6)
Endometriosis	52 (10.2)
Missing data	13 (2.5)
Duration of couple’s infertility [years]	
1–2	39 (7.63)
2–3	141 (27.59)
3–5	151 (29.55)
> 5	180 (35.23)
Alcohol use	
None or < 1 drink/week	281 (55.0)
1–3 drinks /week	224 (44.0)
Everyday	6 (1)

The mean (±SD) value for antral follicle count was 12.73 ± 8.94. The mean level of reproductive hormones were 1.17 ± 1.46 ng/ml for AMH, 6.38 ± 2.18 IU/l for FSH and 93.74 ±16.63 pg/ml for E_2_ (Table 2).

**Table 2:** Ovarian reserve parameters among study population

Parameters	A Mean ± SD	G Mean ± SD	Min	Q25	Median	Q75	Q95	Max
AFC (n)	12.73 ± 8.94	12.25 ± 1.73	1	8	11	20	30	40
AMH (ng/ml)	1.17 ± 1.46	1.21 ± 1.4	0.02	0.9	1.3	2.9	9.36	18
FSH (IU/l)	6.38 ± 2.18	6.00 ± 1.43	0.9	4.86	6.14	7.51	10.48	13.5
E2 (pg/ml)	93.74 ± 16.63	91.33 ± 12.89	75	83	95	120	180	200

*A Mean* aritmetic mean, *G Mean* geometric mean, *SD* standard deviation, *Min* minimal value, *Max* maximum value, *Q25* 25 perentyl, *Q75* 75 percentyl, *Q95* 95 percentyl, *AMH* Anti-Müllerian hormone, *AFC* antral follicle count, *FSH* follicle-stimulating hormone, *E2* estradiol

### Parabens levels in urine

Table 3 summarizes the unadjusted and adjusted urinary parabens concentrations in the first and second urine samples. In the first sample the parabens with the highest geometric mean concentration was MP (92.68 μg/L, 107.93 μg/L SG-adjusted) followed by PP (16.20 μg/L, 18.67 μg/L SG-adjusted), EP (11.28 μg/L, 12.90 μg/L SG-adjusted), BP (4.70 μg/L, 5.02 μg/L SG-adjusted) and iBuP (3.16 μg/L, 2.80 μg/L SG-adjusted).

**Table 3:** Distribution of urinary parabens concentration in urine samples

Parabens in urine (μg/L)	Statistics								
	A Mean ± SD	G Mean ± SD	LOD	Q25	Median	Q75	Q95	Max	>LOD (%)
First urine sample N = 511									
MP	198.91 ± 224.37	92.68 ± 4.28	0.5	37.69	119.74	280.89	688.12	1260.36	93.92
EP	57.21 ± 112.02	11.28 ± 7.00	0.5	2.00	9.52	56.42	258.96	785.72	84.31
PP	97.80 ± 430.61	16.20 ± 6.33	0.5	4.06	15.54	59.21	371.12	7193.09	84.11
BP	10.13 ± 20.22	4.70 ± 2.96	1.0	1.99	3.95	8.60	40.89	202.43	64.12
iBuP	5.19 ± 6.53	3.16 ± 2.55	1.0	1.35	2.47	6.07	19.06	34.95	10.60
∑ Parabens (nmol/ml)	176.61 ± 158.75	25.60 ± 4.62	0.5	9.42	30.24	82.24	275.63	1895.31	
SG adjusted (μg/L)									
MP	232.97 ± 287.21	107.93 ± 4.39	0.5	43.1	145.76	316.12	731.34	2922.30	93.92
EP	62.64 ± 121.45	12.90 ± 7.04	0.5	2.37	13.10	62.76	264.13	42.43	84.31
PP	98.94 ± 382.95	18.67 ± 6.25	0.5	4.56	18.49	72.70	359.68	5394.81	84.11
BP	10.90 ± 21.58	5.02 ± 3.05	1.0	2.27	4.29	9.73	37.21	197.77	64.12
iBuP	4.61 ± 5.54	2.80 ± 2.68	1.0	1.24	2.53	4.86	15.38	30.92	10.60
Second urine sample N = 120									
MP	116.62 ± 154.42	49.13 ± 105.39	0.5	19.08	61.22	154.14	216.85	993.87	90.83
EP	33.91 ± 71.06	5.71 ± 44.97	0.5	1.19	3.51	24.09	37.21	391.51	71.67
PP	32.91 ± 71.06	9.14 ± 38.58	0.5	2.44	9.86	25.65	42.11	504.93	70
BP	9.44 ± 30.90	3.99 ± 9.90	1.0	1.90	3.43	7.76	9.21	235.34	47.5
∑ Parabens (nmol/ml)	48.22 ± 81.86	16.99 ± 198.84	0.5	6.16	4.88	52.91	76.34	531.41	
SG adjusted (μg/L)									
MP	149.84 ± 207.56	67.18 ± 125.60	0.5	24.20	91.16	204.18	312.78	1715.13	90.83
EP	38.45 ± 77.20	7.85 ± 8.85	0.5	1.58	4.73	37.71	42.15	534.96	71.67
PP	39.75 ± 82.17	46.49 ± 52.13	0.5	3.46	11.49	10.02	17.21	470.18	70
BP	8.95 ± 17.41	4.74 ± 7.73	1.0	2.23	4.37	9.51	11.22	126.08	47.5

*A Mean* artimetic mean, *G Mean* geometric mean, *Q25* 25 quartile, *Q75* 75 quartile, *Q95* 95 quartile, *LOD* limit of detection, *MP* methyl-paraben, *EP* ethyl-paraben, *PP* propyl-paraben, *BP* butyl-paraben, *iBuP* izobuthyl-paraben

The second urine sample was collected from 120 women. The highest geometric mean concentration was MP (49.13 μg/L, 67.18 μg/L SG-adjusted) followed by PP (9.14 μg/L, 46.49 μg/L SG-adjusted), EP (5.71 μg/L, 7.85 μg/L SG-adjusted), BP (3.99 μg/L, 4.74 μg/L SG-adjusted). iBuP was not detected in the second urine samples (Table 4).

**Table 4:** Spearman correlation between parabens

First urine sample (***n*** = 511)					
	MP	EP	PP	iBuP	BP
MP r (p)	1				
EP r (p)	0.55 (**< 0.001)**	1			
PPr (p)	0.64 (**< 0.001)**	0.41 (**< 0.001)**	1		
iBuPr (p)	0.13 (**0.002)**	0.17 (**< 0.001)**	0.16 (**< 0.001)**	1	
BPr (p)	0.30 (**< 0.001)**	0.28 (**< 0.001)**	0.28 (**< 0.001)**	0.34 (**< 0.001)**	1
**Second urine sample (*N*** **= 120)**					
	MP	EP	PP	BP	
MP r (p)	1				
EP r (p)	0.44 (**< 0.001)**	1			
PP r (p)	0.49 (**< 0.001)**	0.37 (**< 0.001)**	1		
BP r (p)	0.26 (**0.004)**	0.16 (0.09)	0.13 (0.17)	1	

Note: *p p* values, *r* correlations coefficient, *MP* methyl-paraben, *EP* ethyl-paraben, *PP* propyl-paraben, *BP* butyl-paraben, *iBuP* izobuthyl-paraben

Examined parabens were highly correlated in the first urine samples: MP with EP, PP, BP (*p* < 0.001) and with iBuP (*p* = 0.002). EP was significantly correlated with PP, BP, iBuP (*p* < 0.001), PP with iBuP and BP (*p* < 0.001) and BP with iBuP (*p* < 0.001). In the second urine sample the MP was highly correlated with EP, PP (*p* < 0.001) and BP (*p* = 0.004). EP was significantly correlated with PP (*p* < 0.001). Whereas no statistically significant correlations was observed between EP and BP (*p* = 0.09) and PP and BP (*p* = 0.17) (Table 4).

There was a significant correlation between MP, EP and PP in the first and second urine sample (Table 5). Only in case of BP there was not significant correlation in both urine samples.

**Table 5:** Correlations between parabens in two urine samples

Parabens	Coef (S1-S2)	p (S1-S2)
MP	0.31	**0.001**
EP	0.32	**< 0.001**
PP	0.39	**< 0.001**
iBuP	No detected in the second urine sample	
BP	0.08	0.39

*MP* methyl-paraben, *EP* ethyl-paraben, *PP* propyl-paraben, *BP* butyl-paraben, *iBuP* izobuthyl-paraben, *S1* first urine sample, *S2* second urine sample

### Urinary parabens concentration and ovarian reserve

In multivariate linear regression models where the exposure variable was treated as a continuous variable only PP decrease AFC (*p* = 0.04), E_2_ level (*p* = 0.04) and increase the FSH concentration (*p* = 0.028) (Table 6). MP, EP, BP and iBuP were not associated any with parameters of ovarian reserve (AFC and the concentrations of AMH, FSH and E_2_). The models were adjusted for age, smoking, BMI and initial infertility diagnosis.

**Table 6:** The association between parabens and parameters of ovarian reserve- categorical variable

		AFC			AMH			FSH			E2		
	Categories	coef	95%CI	p	coef	95%CI	p	coef	95%CI	p	coef	95%CI	p
MP	Cont.	− 0.02	− 0.06;0.01	0.14	− 0.05	− 0.10;0.001	0.06	− 0.01	− 0.03;0.01	0.51	− 0.01	− 0.05;0.02	0.52
	Q2	0.03	− 0.13;0.18	0.72	0.15	−0.09;0.40	0.22	0.05	− 0.05;0.15	0.34	0.03	− 0.15;0.20	0.76
	Q3	−0.11	− 0.27;0.04	0.16	− 0.11	− 0.36;0.13	0.37	− 0.08	− 0.19;0.03	0.14	0.05	− 0.12;0.23	0.55
	Q4	−0.05	− 0.21;0.11	0.54	−0.16	− 0.41;0.09	0.20	− 0.04	− 0.14;0.07	0.50	− 0.08	− 0.25;0.10	0.39
	*P* for trend	0.52			0.21			0.50			0.40		
EP	Cont.	−0.004	− 0.03;0.02	0.77	− 0.03	− 0.07;0.02	0.22	0.002	− 0.02;0.02	0.81	− 0.03	− 0.06;0.05	0.09
	Q2	−0.01	−0.17;0.15	0.95	−0.15	− 0.41;0.10	0.24	− 0.09	−0.20;0.02	0.12	0.04	−0.14;0.22	0.66
	Q3	0.03	−0.13;0.18	0.76	0.02	−0.23;0.26	0.91	0.01	−0.09;0.12	0.79	−0.01	−0.24;0.11	0.45
	Q4	−0.02	−0.18;0.14	0.83	−0.18	− 0.43;0.07	0.16	− 0.05	−0.15;0.06	0.39	−0.14	− 0.32;0.04	0.12
	*P* for trend	0.84			0.20			0.39			0.29		
PP	Cont.	−0.02	−0.13;-0.01	**0.04**	−0.03	−0.07;0.02	0.22	0.03	0.02;0.12	**0.028**	−0.03	−0.06;-0.001	**0.04**
	Q2	−0.06	−0.22;0.04	0.14	−0.09	− 0.35;0.16	0.49	0.02	−0.08;0.01	0.09	−0.12	− 0.29;0.05	0.18
	Q3	−0.03	−0.20;-0.02	**0.048**	−0.01	− 0.3;0.25	0.96	0.05	0.03;0.18	**0.026**	−0.11	−0.20;-0.01	**0.03**
	Q4	−0.03	−0.18;-0.03	0.05	−0.08	− 0.33;0.17	0.54	0.02	−0.08;0.03	0.07	−0.13	− 0.31;0.04	0.13
	*P* for trend	**0.04**			0.36			**0.03**			**0.04**		
iBuP	Cont.	0.02	−0.05;0.09	0.61	−0.003	−0.11;0.10	0.95	−0.02	− 0.07;0.02	0.31	− 0.02	−0.10;0.06	0.62
	>LOD	0.1	−0.07;0.27	0.24	0.03	−0.24;0.30	0.84	−0.01	−0.11;0.10	0.96	−0.13	− 0.31;0.04	0.14
BP	Cont.	0.004	−0.04;0.05	0.84	−0.05	−0.12;0.01	0.11	−0.01	− 0.04;0.02	0.49	0.005	−0.04;0.05	0.83
	Q2	−0.04	−0.19;0.12	0.64	−0.02	− 0.26;0.22	0.87	− 0.03	−0.13;0.07	0.58	−0.13	− 0.30;0.04	0.14
	Q3	0.07	−0.08;0.22	0.35	0.02	−0.23;0.26	0.89	−0.09	−0.19;0.01	0.08	0.05	−0.12;0.22	0.56
	Q4	0.01	−0.14;0.16	0.89	−0.20	−0.45;0.04	0.11	−0.01	− 0.11;0.10	0.90	− 0.08	−0.25;0.09	0.37
	*P* for trend	0.89			0.12			0.90			0.36		

Model adjusted for: age (years), BMI (kg/m^2^), smoking (Yes/No) and initial infertility diagnosis. Parabens exposure in the model was based on the first urine sample

*MP* methyl-paraben, *EP* ethyl-paraben, *PP* propyl-paraben, *BP* butyl-paraben, *iBuP* izobuthyl-paraben

Reference groups 1. In case >LOD reference is <LOD; 2. In case Q2, Q3, Q4 reference is Q1; Cont.-continuous variable

Q1- ≤ 25 percentyl; Q2- (25–50] percentyl; Q3-(50–75] percentyl; Q4- > 75 percentyl

Similar results were observed when the exposure variables were categorized. Exposure to PP in the third quartile (50th -75th] was negatively associated with antral follicle count (*p* = 0.048) and E_2_ concentrations (*p* = 0.03) and positively with FSH (*p* = 0.026) (Table 6).

## Discussion

In the study, we found a relationship between urinary concentrations of parabens and parameters of ovarian reserve. The statistically significant associations were observed between urinary concentrations of PP and decrease AFC, E_2_ level and increase the FSH concentration.

To our knowledge, only one human study has investigated the association between urinary paraben concentrations and female ovarian reserve [13]. Our findings are in line with the study performed by Smith et al. (2013) [13] where a negative relationship between urinary PP and AFC was also found. Higher urinary PP was associated with a higher FSH. In this study PP concentrations in urine were associated with decrease antral follicle count, estradiol level and increased the FSH concentrations. Other examined parabens: MP, EP, BP and iBuP were not associated any with parameters of ovarian reserve (antral follicle count and the concentrations of AMH, FSH and E_2_).

The relationship of PP with diminished ovarian reserve is consistent with animal data showing that the estrogenicity of parabens, and therefore the potential for reproductive toxicity, is greater in PP compared with MP [7, 8, 23]. Although the animal data also show that BP and iBuP is more estrogenic than PP or MP, we detected BP and iBuP less frequently. When the chemical was detected, urinary concentrations of BP were much lower than those of either PP or MP, which may explain the lack of an association of BP with markers of ovarian reserve. It is also possible that biological activity and mechanisms of action differ between the parabens.

The mechanism of the potential impact of nonpersisntant environmental factors such as parabens is associated with the roles of the aryl hydrocarbon receptor (AHR) and the estrogen receptor (ER) systems in ovarian reserve modulation. Environmental compounds are similar to the natural ligands and have the ability to bind to these receptors and have the potential to influence either the initial setting of ovarian reserve during development or the trajectory of ovarian reserve during adult life [24].

One of the predictors of women fertility is undoubtedly ovarian reserve, which could be used to determine the potential of female fecundity. Therefore, it is important that ovarian reserve is looked closely in studies on fertility. AFC is considered one of the best markers of ovarian reserve [25]. Additionally serum AMH level can be assessed regardless of pregnancy-attempts status and the measurements are not significantly affected by phase of menstrual cycle [26]. Baird and Steiner (2012) [26] suggest that this hormone should be investigated as an independent measure of fecundability in studies that focus on exposures hypothesized to target the ovary and is a potential tool in epidemiologic studies of female fertility.

Urinary concentrations of parabens in our study subjects were comparable to those reported in a national sample of US women in the fourth report of the National Health and Nutrition Examination Survey (NHANES) with respect to PP (CDC 2017) [27]. Geometric mean concentrations recently reported in females participating in the NHANES 2011–2014 were 13.5 μg/L compared to 15.54 μg/L in the present study. The median urinary concentrations of MP, EP and BP were higher in our study than in NHANES 119.74 μg/L and 73.9 μg/L for MP, 9.52 μg/L and 1.60 μg/L for EP and 3.95 μg/L and < LOD for BP respectively. Also the concentrations of parabens found in our study were higher than in the study performed among Tunisian women (urinary geometric mean concentrations of MP-30.1 ng/ml, EP-1.4 ng/ml, PP-2.0 ng/ml, BP-0.5 ng/ml) [28]. The urinary concentration of iBuP was not measured in the US survey. The iBuP level was assessed in an urban community of Western Canada where the urinary concentrations were lower (median 0.22 μg/L) than in the present study (median 2.47 μg/L). The differences in the level of iBuP may be associated with the fact that in the study in Canada, the exposure assessment was performed among 11 participants [29].

To the best of our knowledge, this is the largest human study to date to evaluate the association between environmental exposure to parabens and parameters of ovarian reserve. The limitations is the cross-sectional design of this study which does not allow the inference of causal relationships and, consequently, reverse causality issues cannot be ruled out. Due to its design, it may not be possible to generalize our findings to women who are not seeking fertility evaluation. Additionally, we only have considered paraben exposure, but women are exposed to a wide range of environmental chemicals that may contribute to the mixture effect.

The strength of our study is the collection which include two urine samples. Although one urine sample may reasonably represent several months of exposure, collection of multiple samples should reduce exposure misclassification during the time period.

In addition, in the present study, all subjects were recruited in the same center, and all samples were collected and analyzed using a standardized protocol. Detailed questionnaire on demographics, medical and lifestyle risk factors received from the participants allowed for control of confounding variables in the statistical models. Since the present study was conducted among women recruited through an infertility clinic, it may limit the ability to generalize the results to the general population. On the other hand these findings may be of concern because of increased use of parabens that results in widespread exposure among the general population and lack of human epidemiologic studies. Further epidemiological studies will be helpful to better understand the effects of parabens on female fecundity.

In conclusion, we observed an inverse association between urinary concentrations of PP and decrease AFC and E_2_ level. Additionally PP was positively associated with FSH in women attending a fertility center. Our findings could be suggestive that a chronic exposure to PP may potentially contributing to reduced fecundity and impair fertility. As this is one of the first study to investigate the potential effect of parabens on ovarian reserve further epidemiological studies with longer duration of observation are needed.

## Data Availability

The datasets used and analyzed during the current study are available from the corresponding author on reasonable request.
